# Assessment of the effectiveness and efficiency of the economic community of West African States Medicines Regulatory Harmonization initiative by the pharmaceutical industry

**DOI:** 10.3389/fphar.2023.1184108

**Published:** 2023-05-09

**Authors:** Mercy Owusu-Asante, Delese Mimi Darko, Stuart Walker, Sam Salek

**Affiliations:** ^1^ School of Life and Medical Sciences, University of Hertfordshire, Hatfield, United Kingdom; ^2^ Food and Drugs Authority, Accra, Ghana; ^3^ Centre for Innovation in Regulatory Science, London, United Kingdom; ^4^ Institute of Medicines Development, Cardiff, United Kingdom

**Keywords:** effectiveness, efficiency, West Africa Medicines Regulatory Harmonization (WA-MRH), joint assessment procedure, benefits

## Abstract

**Background:** Following the establishment of Economic Community of West African States Medicines Regulatory Harmonization (ECOWAS-MRH) initiative in 2017, it was considered timely to carry out an evaluation of the current status of the initiative’s operating model by the pharmaceutical industry users. This study examined the challenges being encountered and identified strategies that would strengthen the ECOWAS-MRH initiative moving forward.

**Methods:** The Process Effectiveness and Efficiency Rating (PEER) questionnaire was used to collect data from manufacturers who have submitted applications to the joint assessment procedure and had identified recommendations for improving the performance of the ECOWAS-MRH initiative.

**Results:** Ten pharmaceutical manufacturer participants (innovator, generic foreign, generic local) all reported that harmonisation of registration requirements was a major benefit, allowing submission of the same dossier to multiple countries, reducing the application burden and saving time and resources. Additionally, receipt of the same list of questions from several countries enables the compilation of a single response package, resulting in shorter timelines for approvals compared to the individualised country responses. Another benefit of a harmonised registration process was the simultaneous accessibility of medicines in various markets. Key challenges included a lack of centralised submission and tracking, differences in regulatory performance of the national medical regulatory authorities, a lack of detailed information for applicants and a low motivation to use the ECOWAS-MRH route with a preference for other regulatory pathways in the ECOWAS member states.

**Conclusion:** This study identified several approaches to increase the effectiveness of this initiative: the implementation of risk-based approaches such as use of reliance pathways; establishment of a robust information technology systems, building assessor capacity to facilitate processing and monitoring applications; and priority review of ECOWAS-MRH products.

## 1 Introduction

It is the responsibility of every country to establish an effective and efficient medicine regulatory system in order to ensure timely patients’ access to high-quality, safe and efficacious medicines (WHO, 2014). Vogel (2002) noted that “…until recently, drug regulation was virtually synonymous with national sovereignty”. Globally, the regulation of medicines is comparatively stringent when compared with the regulation of other consumables and to achieve an effective and efficient medicine regulatory system requires the involvement of multiple stakeholders, notably, the manufacturers of medicines and vaccines (Roth et al., 2018).

The active engagement and co-operation of manufacturers with regulators in the medicine regulatory system is vital to the success of a national health system. Presently, there are multiple national jurisdictions that manufacturers have to contend with in order to obtain the requisite raw or starting materials, intermediate or semi-finished products, and where applicable, packaging materials from one country into another so as to manufacture a finished pharmaceutical product or vaccine. Furthermore, there are multinational pharmaceutical companies who conduct activities such as contract manufacturing across multiple countries worldwide, which underlines the complex nature of manufacturing of medicines and vaccines. The ideal regulatory environment that would benefit applicants would be one that multiple countries subscribe to under an umbrella of harmonisation. Furthermore, there is evidence to show that pharmaceutical applicants have a selective bias with regard to regulatory systems that provide transparency, accountability and predictability (Vogel, 2002; Lakkis, 2010; Preston et al., 2012; Sillo et al., 2020).

There are various regulatory mechanisms available at this time that correspond to these parameters, such as the well-established centralised authorisation procedure of the European Union. In this procedure, applicants apply for a single centralised marketing authorisation for a product, which is valid throughout the European Union member states as well as Iceland, Liechtenstein and Norway (European Medicines Agency, 2022). Similarly, there are Medicine Regulatory Harmonisation (MRH) initiatives such as those of the World Health Organization (WHO) that meet expectations of stakeholders and encourage applicants to submit marketing authorisation applications in other regions of the world (WHO, 2008; Lakkis, 2010; Ncube et al., 2021).

Presently, various diseases such as malaria and tuberculosis occur predominantly in Africa, with the WHO stating that in 2017, 92% of the 219 million cases of malaria reported worldwide were from Africa (WHO, 2022a); therefore, after many years of using well-established medicines (generics), it is worth recommending that to obtain better patient outcomes, new medicines and vaccines should be developed by manufacturers primarily in Africa for use by the African population.

The challenges to the assessment of applications for marketing authorisation for new chemical entities and vaccines in Africa have been identified (WHO, 2008). Moran and colleagues have published a number of mechanisms that could be explored to meet these challenges; notable among these are to establish an ideal drug regulatory system in Africa and enhance the regulatory capacity of assessors to make it possible for them to assess the quality, safety and efficacy data for new medicines to be used in Africa to a high standard and in a timely manner (Moran M et al., 2011; Roth L et al., 2018).

Medicine regulatory harmonisation appears to be an effective mechanism for deploying technical, human and financial resources efficiently for the benefit of the population. According to the WHO, applicants who participate in the WHO prequalification programme enjoy various benefits such as increased sales or market access, improved image or brand, reduced manufacturing costs and increased capacity/skills. By extrapolation, applicants who participate in similar harmonisation initiatives like the ECOWAS-MRH can also experience their share of these benefits by having access to patients in all the 15 member states of ECOWAS, making this a win-win situation for both the applicants and patients (WHO, 2022b).

Started in 2017 by the West African Health Organization (WAHO), the ECOWAS-MRH initiative seeks to enhanced accessibility of high-quality, safe and effective medicines and vaccines in the ECOWAS (Owusu-Asante, 2022). By the joint registration of both local and imported medical products, this programme seeks to expedite registration and advance regulatory processes (Daniel, 2019). As of 2023, seven NMRAs take part in initiative sessions: Burkina Faso, Cote d’Ivoire, Ghana, Nigeria, Senegal, Sierra Leone and Togo. Whilst only these seven countries take part in the joint assessments, the results of the assessments form the foundation for regulatory decisions in all 15 ECOWAS NMRAs (Owusu-Asante, 2022). Using a rotational system, the ECOWAS-MRH appoints one country to act as lead NMRA/coordinator for 2 years and acts as the coordinator for product applications. This country validates and prepares applications for review by a group of assessors from the seven participating NMRAs and their report is evaluated by the expert working group in a joint assessment session. The WAHO Secretariat then issues notifications of recommendations at the regional level and finally, NMRAs apply their own national procedure to issue a national marketing authorisation to applications for a jointly reviewed product.

About a decade ago, Narsai and colleagues reported that there was inadequate information in the literature detailing the views of pharmaceutical manufacturers about the regulatory systems in Africa (Narsai et al., 2012). This situation has now seen some improvement, following studies published in 2022 by Sithole and colleagues with reference to the ZaZiBona initiative (Sithole et al., 2022) and also by Ngum and colleagues regarding the East African Community initiative (Ngum et al., 2022). More studies should therefore be conducted and published so that much-needed data become available to all stakeholders. Additionally, opportunities for a better alignment between industry and regulators should be pursued and “the fact should not be forgotten that access to medicines on time for everyone is a human right rather than a luxury.” (Oge, 2020).

As a result of completing an earlier study aimed to assess the effectiveness and efficiency of the current operating model of the ECOWAS-MRH initiative by the national medical regulatory authorities (NMRAs) in the member countries (Owusu-Asante et al., 2022), this present study aimed to assess the effectiveness and efficiency of the ECOWAS-MRH initiative by the pharmaceutical industry in order to obtain a holistic view of the current status of the initiative.

The objectives of this study were to obtain the views of the pharmaceutical applicants or their local representatives of the ECOWAS-MRH initiative about the performance of the programme to date, identify the challenges experienced by the applicants throughout the life cycle of the ECOWAS-MRH initiative, determine the strengths and weaknesses of the initiative, identify the ways of improving the performance of the work-sharing programme and envisaging the strategy for moving forward.

## 2 Materials and methods

### 2.1 Study participants

All ten pharmaceutical manufacturers who have submitted marketing authorisation applications for assessment of medicines at the regional level since the beginning of the ECOWAS-MRH initiative participated in the study. The study participants were classified as Innovator, Generics (foreign) -manufacturer outside ECOWAS and Generics (local)- manufacturer within ECOWAS.

### 2.2 Data collection

Data for the study were obtained through completion of the Process Effectiveness and Efficiency Rating (PEER) questionnaire (Ngum et al., 2022; Sithole et al., 2022) by applicants between October 2022 and January 2023.

The questionnaire consists of five sections namely; demographics, the benefits and challenges of the ECOWAS-MRH initiative, improving the performance (effectiveness and efficiency) of the work-sharing programme and envisaging the strategy for moving forward.

Semi-structured interviews with a checklist were conducted with each manufacturer in order to validate and elaborate their responses in the PEER questionnaire. This also provided the study participants with an opportunity to discuss any difficulty they faced in responding to some of the sections of the questionnaire. In addition, this post-completion of the questionnaire was designed for the applicants to reflect both their experience of the initiative and that of the ECOWAS-MRH through a dialogue.

## 3 Results

For the purpose of clarity, the results of the study are presented in five parts: 1) Demographics; 2) Benefits of the ECOWAS-MRH initiative; 3) Challenges of the ECOWAS-MRH initiative; 4) Improving the performance (effectiveness and efficiency) of the work-sharing programme; and 5) Envisaging the strategy for moving forward.

### 3.1 Part 1. Demographics

The respondents were mostly regulatory affairs managers, with varying years of regulatory experience ranging from 3 to 30. A summary of the manufacturers and their product categories is provided in Table 1. There was only one respondent from each company and all those companies that had started to use the procedure were included in the sample size regardless of whether their product was approved or deferred.

**TABLE 1:** Pharmaceutical applicants participating in study.

Name of applicant	Innovator	Foreign generic	Local generic	Number of submissions	Therapeutic category	Status
Drugfield Pharmaceuticals Ltd			√	1	Disinfectant & antiseptic	Approved
Cipla Quality Chemical Industries Ltd		√		4	Antiretroviral (4)	Approved (3)
						Deferred (1)
Pfizer Specialties Ltd	√			1	Biological product	Approved
Mission Pharma A/S, Denmark		√		1	Reproductive health product	Approved
Novartis Pharma AG		√		1	Biological product	Approved
Emzor Pharmaceutical Industries Ltd			√	5	Antidiarrhoeal Antimalarial (2)	Approved (1)
					Disinfectant& antiseptic	Deferred (1)
					Reproductive health product	Screened (3)
Juhel Nigeria Ltd			√	1	Antifungal	Deferred
May & Baker			√	3	Antibacterial	Screened (3)
					Antimalarial	
					Antiretroviral	
M& G Pharmaceuticals Ltd			√	2	Antidiarrhoeal (2)	Screened (2)
Laurus Laboratories Ltd		√		1	Antiretroviral	Screened (1)

Eight out of the 20 submissions have been issued with recommendation letters. The remaining submissions have either been deferred or are still in the screening phase for various reasons.

**FIGURE 1:**
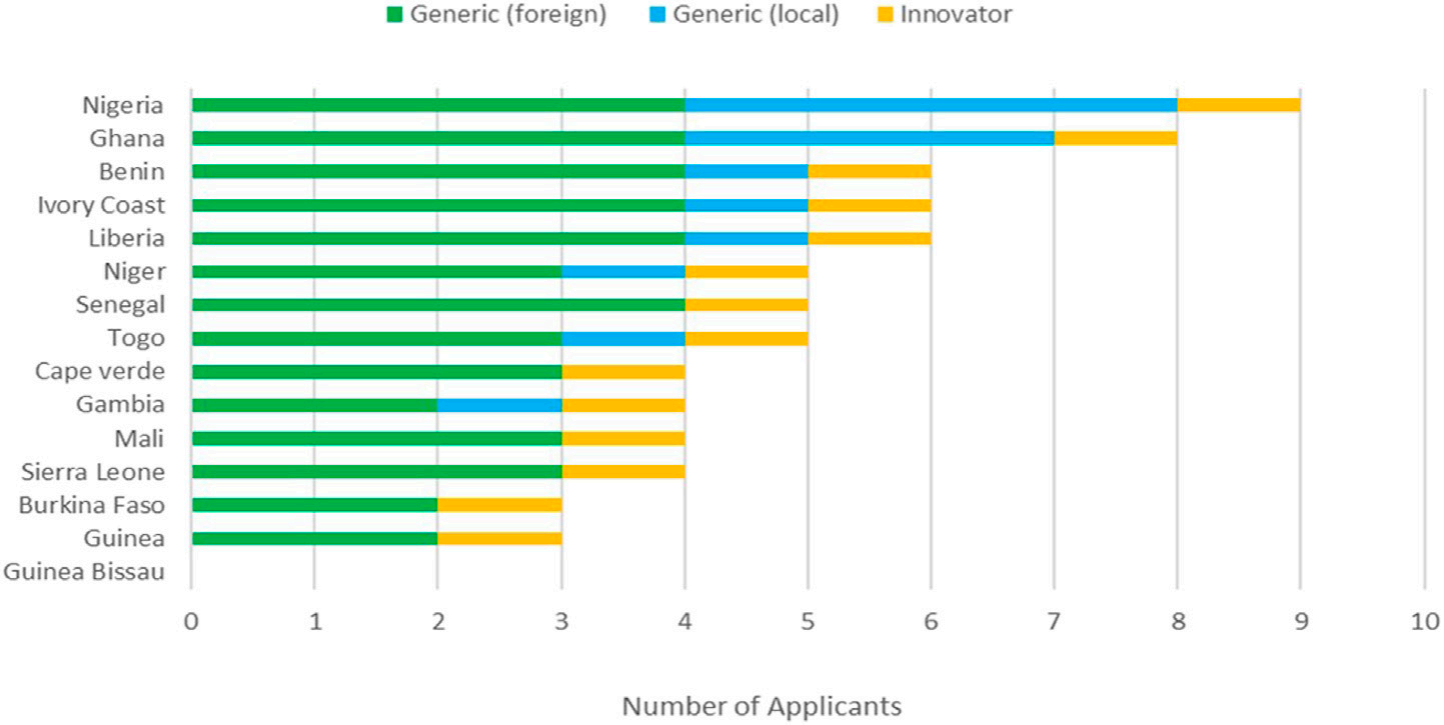
Availability of products in ECOWAS countries.

Both the innovator and foreign generics are available in almost all the ECOWAS member states with the exception of Guinea Bissau; however, local generics are mostly available in Nigeria and Ghana (Figure 1). The assumption therefore has been made that innovative products are not manufactured in the region, but generics are.

Half of the number of manufacturers (that is, Cipla Quality Chemical Industries Ltd-Foreign generics, Juhel Nigeria Ltd-Local generics, Novartis Pharma AG- Foreign generics, May & Baker—Local generics, M& G Pharmaceuticals Ltd-Local generics) keep a separate record of applications submitted for assessment under the ECOWAS-MRH initiative to facilitate tracking and adherence to deadlines.

### 3.2 Part 2. Benefits of the ECOWAS-MRH initiative

**FIGURE 2:**
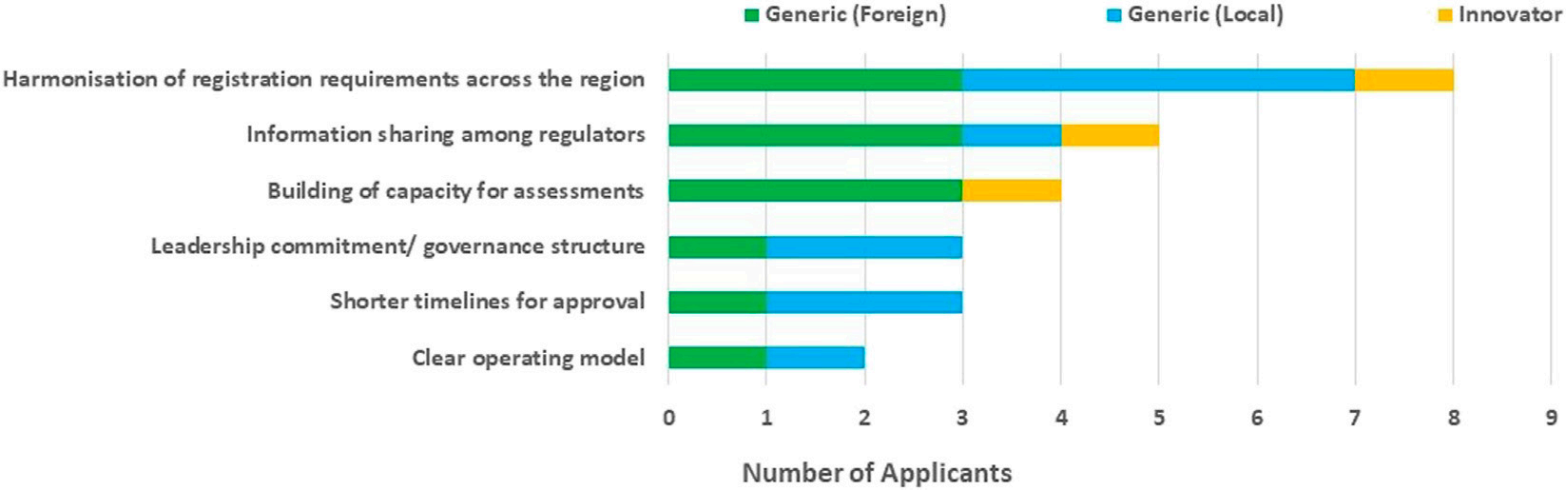
Benefits of the ECOWAS-MRH initiative.

The benefits of the ECOWAS-MRH initiative that were identified by most of the applicants were the harmonisation of registration requirements across the region (80%), information sharing among regulators (50%) and capacity building (40%) for assessments. However, the benefits of leadership commitment/governance structure (30%) and shorter timelines for approval (30%) were identified by a few of the respondents (Figure 2).

#### 3.2.1 Strengths of the ECOWAS-MRH process for recommending the registration of products

The strengths of the ECOWAS-MRH process for recommending the registration of products were identified as priority review of ECOWAS-MRH products, regular committee meetings enabling timely finalisation of products after ECOWAS-MRH recommendation, separate register and tracking of ECOWAS-MRH products and products approved under ECOWAS-MRH made available on each country’s website. Medicines and vaccines eligible for the ECOWAS-MRH joint assessment are those included in the WHO Essential Medicine list, HIV/AIDS, malaria, tuberculosis, reproductive health, neglected tropical diseases, antibiotics, for public health emergencies, registered by stringent regulatory authorities, prequalified by WHO, registered under Swissmedic procedure for scientific advice and Marketing Authorisation for Global Health Products (MAGHP), granted a scientific opinion in line with the European Medicines Agency’s Article 58 of Regulation (EC) No 726/2004, life-saving commodities by the UN Commission on life saving medicines for women and children and other priority medicinal products as determined by the WAHO.

Upon completion of the ECOWAS-MRH joint assessment for such eligible medicines and vaccines, these are then granted marketing authorisation via the national registration system in the relevant country (WA-MRH, 2021; European Medicines Agency, 2023). Priority review of such applications at the regional level therefore, facilitates quicker access to these medicines and vaccines by patients in the ECOWAS region.

#### 3.2.2 Benefits of the ECOWAS-MRH initiative to applicants

**FIGURE 3:**
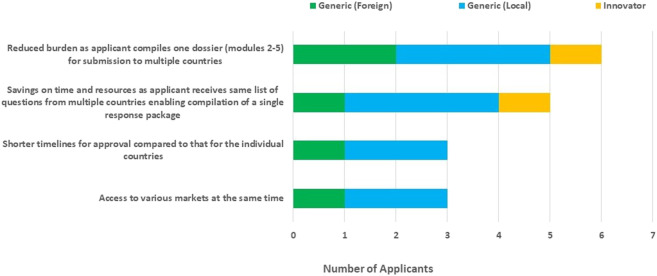
Benefits of the ECOWAS-MRH initiative to applicants.

The benefits of the ECOWAS-MRH initiative identified by applicants (manufacturers) included: a reduced submission burden as applicants compile one dossier (modules 2–5) for submission to multiple countries; savings in time and resources as applicants receive same list of questions from multiple countries, enabling the compilation of a single response package; shorter timelines for approval compared with those for the individual countries and access to various markets at the same time. A better understanding of the individual country requirements was stated as an additional benefit of the ECOWAS-MRH initiative to applicants (Figure 3).

#### 3.2.3 Benefits of the ECOWAS-MRH initiative to patients at the country or regional level

Increased availability of medicines and quicker access to quality assured medicines were reported as benefits of the ECOWAS-MRH initiative to patients in the individual or in the ECOWAS region by the applicants.

### 3.3 Part 3. Challenges of the ECOWAS-MRH initiative

The lack of ability to mandate central registration; differences in the regulatory performance of the countries; dependence on the countries’ processes for communication with applicants; absence of centralised submission and tracking and lack of detailed information on the process for applicants were identified as challenges of the ECOWAS-MRH initiative.

**FIGURE 4:**
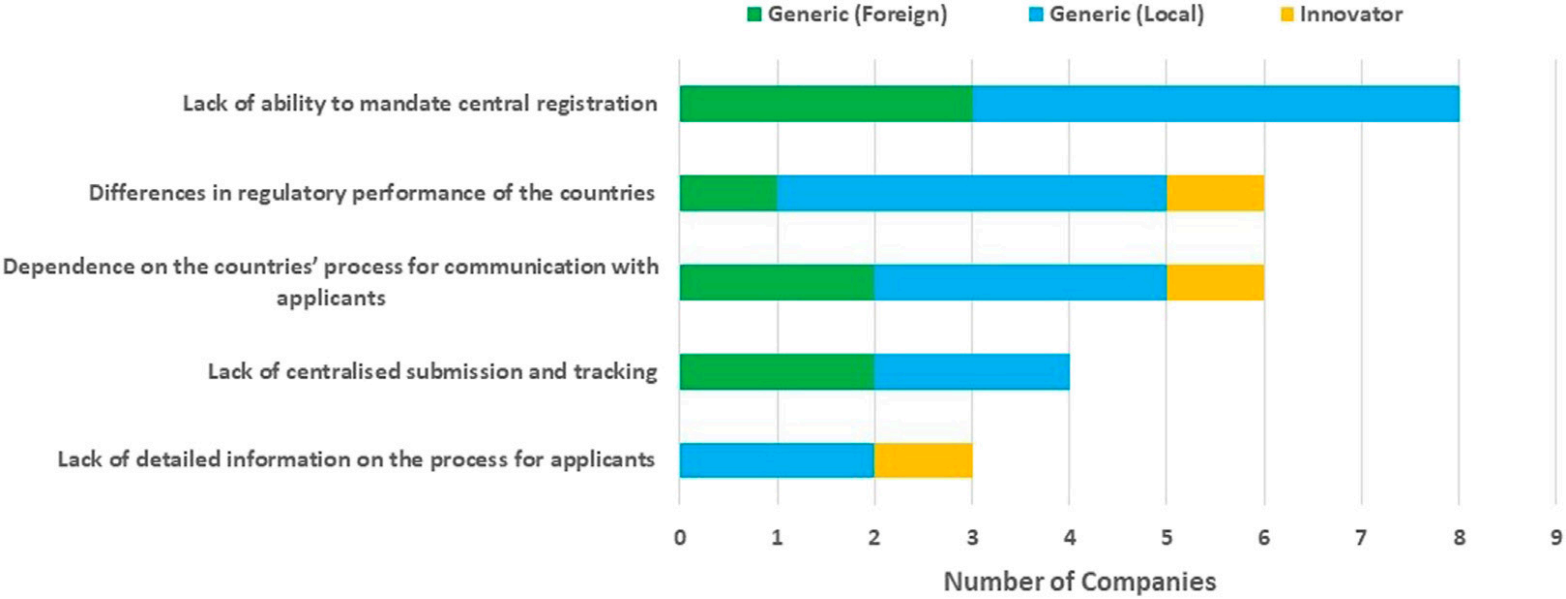
Challenges of the ECOWAS-MRH initiative.

Other challenges were also cited namely, lack of identified NMRA processes for movement from regional to local approval in the ECOWAS countries, no clear process for approval and lack of NMRA responsiveness in communicating updates of application status (Figure 4).

#### 3.3.1 Challenges faced by applicants submitting applications to the ECOWAS-MRH initiative

**FIGURE 5:**
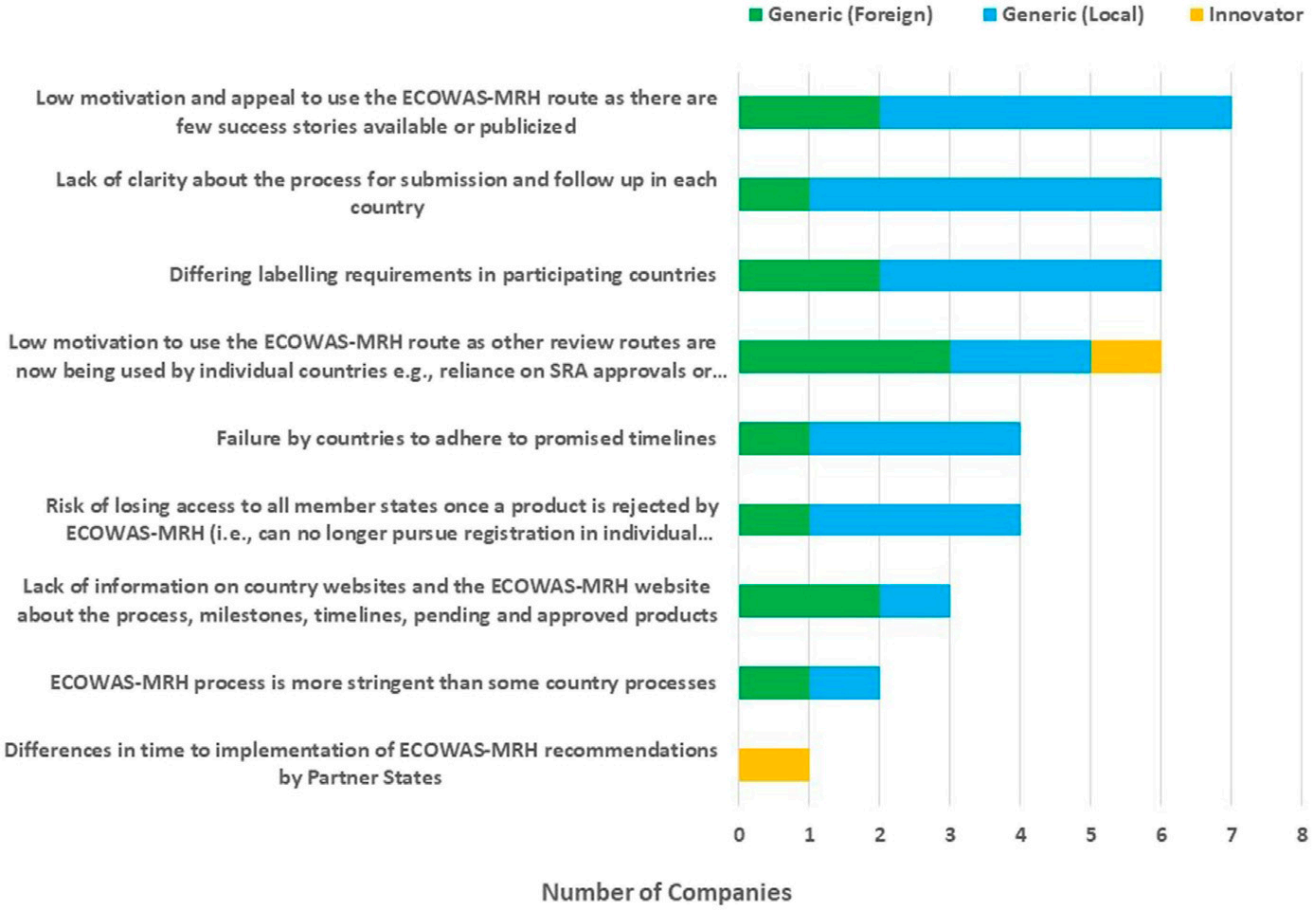
Challenges faced by applicants submitting applications to the ECOWAS-MRH initiative.

Additional challenges identified by manufacturers submitting applications to the ECOWAS-MRH initiative included: low motivation and appeal to use the ECOWAS-MRH route as there are few success stories available or publicised; lack of clarity about the process for submission and follow-up in each country; differing labelling requirements in participating countries and low motivation to use the ECOWAS-MRH route because other review routes such as reliance on approvals from stringent regulatory authority or other ECOWAS countries now being used by individual countries are faster; failure by countries to adhere to promised timelines; the risk of losing access to all member states once a product is rejected by ECOWAS-MRH (that is, applicants can no longer pursue registration in individual countries); a lack of information on country and ECOWAS-MRH websites about the process, milestones or timelines; the absence of lists of pending and approved products; the perception that the ECOWAS-MRH process is more stringent than some country processes; and differences in time to implementation of ECOWAS-MRH recommendations by partner states (Figure 5).

#### 3.3.2 Challenges faced by regulatory authorities in reviewing the ECOWAS-MRH applications

Comments by applicants to the challenges faced by the NMRAs in reviewing the ECOWAS-MRH applications were related to personnel and resources as well as the application and review process as below:

• Lack of enough personnel
• Absence of proper knowledge of review process
• Unharmonised system of review
• Lack of information on country and the ECOWAS-MRH websites about the process, milestones and timelines for pending and approved products
• Each member country has different requirements for Module 1 and applicants having to fulfil all countries’ requirements
• There is no clear process to follow once approval has been received from the ECOWAS- MRH procedure
• Having to bring all the member countries regulators together in a timely manner to review the dossiers/applications
• Delay in response time between applicants and reviewers

### 3.4 Part 4. Improving the performance (effectiveness and efficiency) of the work-sharing programme

#### 3.4.1 Ways to improve the effectiveness of the ECOWAS-MRH initiative

**FIGURE 6:**
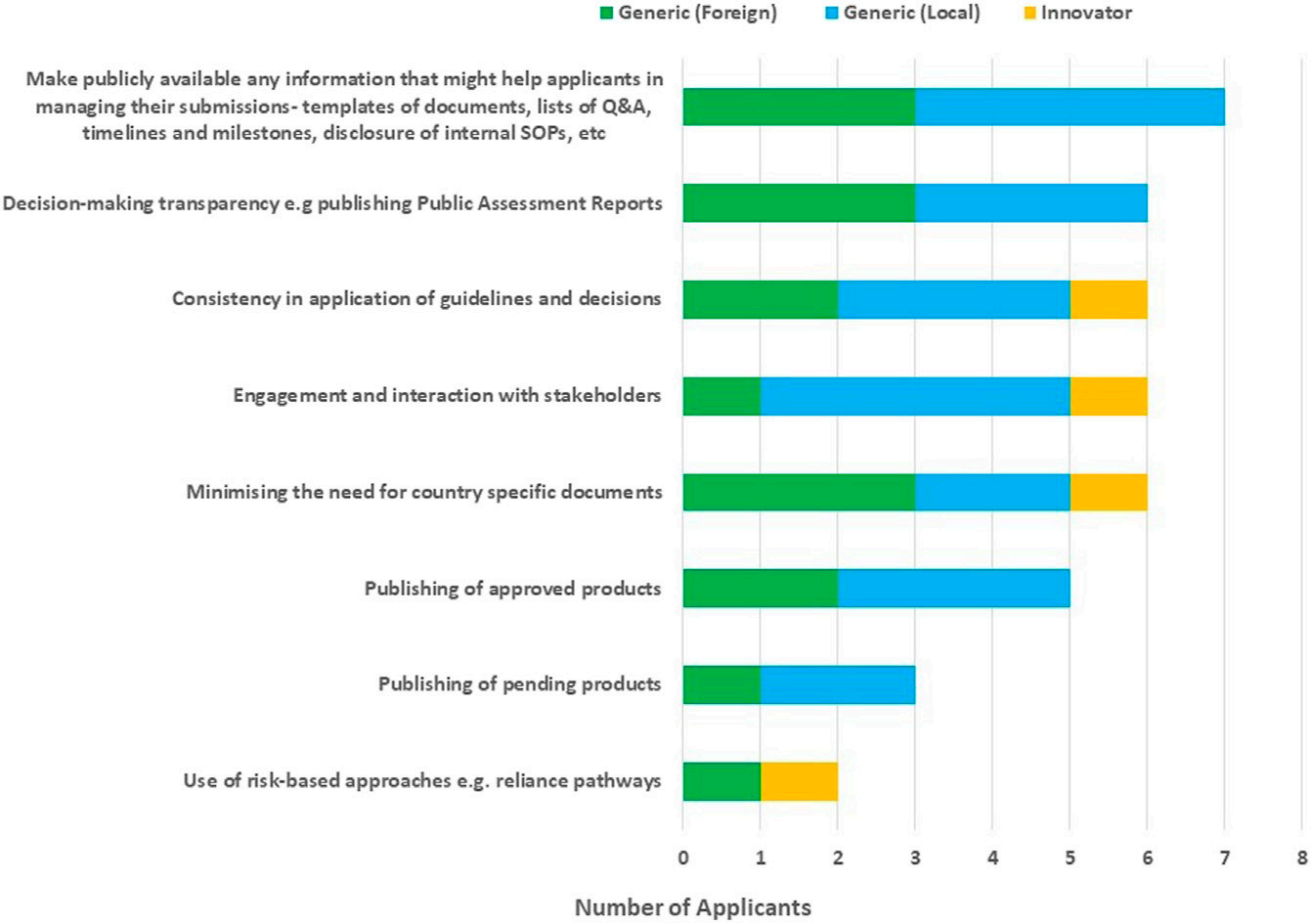
Ways to improve the effectiveness of the ECOWAS-MRH initiative.

Suggestions from applicants to improve the effectiveness of the ECOWAS-MRH initiative (Figure 6) were to make publicly available any information that might help applicants in managing their submissions such as document templates, lists of questions and answers, and timelines and milestones; disclosure of internal standard operating procedures (SOPs); decision/making transparency (for example, publishing Public Assessment Reports); consistency in application of guidelines and decisions, engagement and interaction with stakeholders; minimising the need for country-specific documents; publishing lists of pending and approved products; and the use of risk-based approaches such as reliance pathways.

#### 3.4.2 Ways to improve the efficiency of the ECOWAS-MRH initiative

**FIGURE 7:**
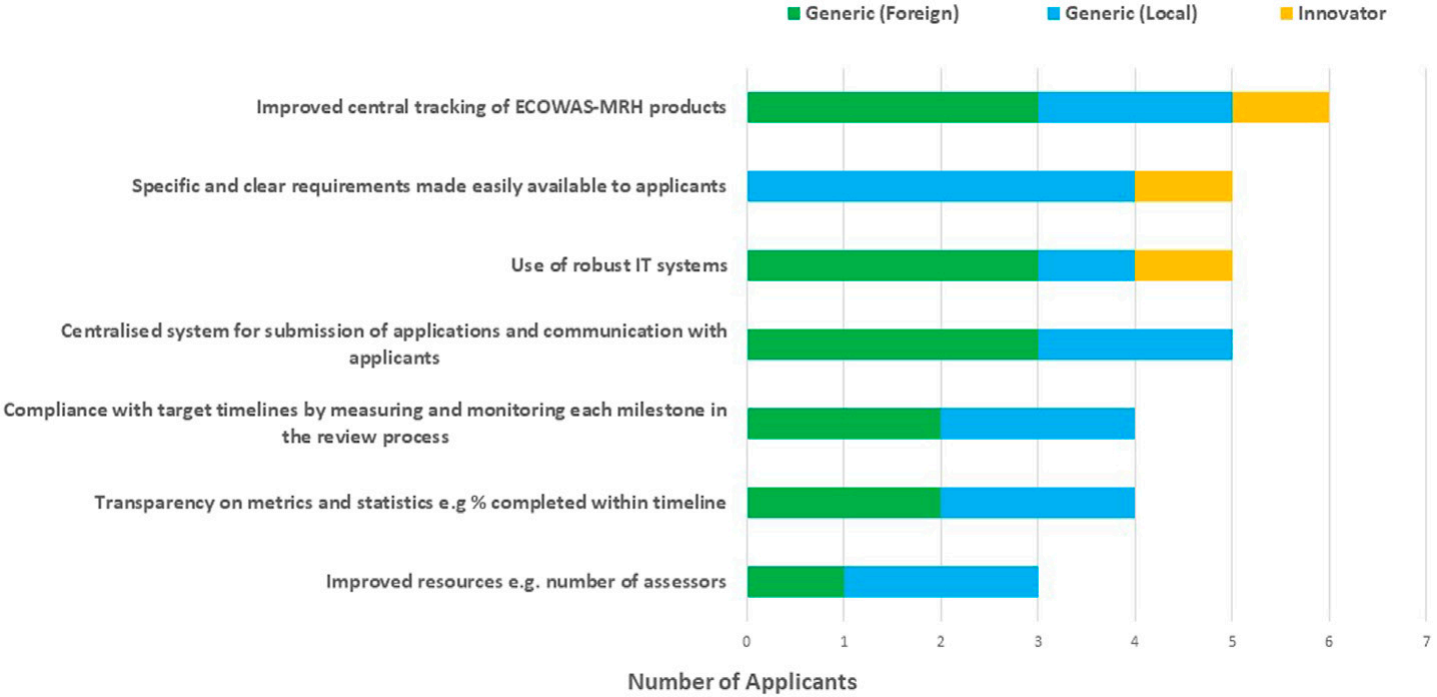
Ways to improve the efficiency of the ECOWAS-MRH initiative.

The applicants suggested the following ways to improve the efficiency of the ECOWAS-MRH initiative: improved central tracking of ECOWAS-MRH products; specific and clear requirements made easily available to applicants; use of robust information technology (IT) systems; a centralised system for submission of applications and communication with applicants; compliance with target timelines by measuring and monitoring each milestone in the review process; and transparency in metrics and statistics such as percentage of reviews completed within timelines; and increased resources such as the number of assessors (Figure 7).

### 3.5 Part 5. Strategy for moving forward

Regarding possible strategies for moving forward, the applicants proposed that the establishment of a regional administrative body to centrally receive and track ECOWAS-MRH applications which would be responsible for allocating work, apportioning the applicable fees to countries, tracking of applications and communication with applicants was the most effective strategy to improve efficiency of the ECOWAS-MRH initiative. On the other hand, continuing with the current operation of the model without any changes was considered the least effective strategy to improve efficiency of the ECOWAS-MRH initiative. Seventy percent (70%) of the respondents agreed that the establishment of an ECOWAS regional medicines agency, if legally possible, will be the best strategy for improved performance going forward.

Finally, the respondents provided strategies that could be considered in strengthening the ECOWAS-MRH initiative going forward.

• Implement a one-time payment process by ECOWAS, eliminating individual NMRA separate statutory registration fees
• Harmonise labelling across all ECOWAS countries, eliminating individual country-specific labelling
• Standardise and harmonise Module 1 dossier requirement across the ECOWAS countries to expedite the registration process
• Require one Certificate of Pharmaceutical Product (CPP) for any of the member countries or for countries where commercialisation is being planned rather than separate CPPs for each country
• Allow applicants to market their products across ECOWAS countries after ECOWAS-MRH approval to expedite the availability of medicines
• Provide an online ECOWAS-MRH portal application system to expedite application submissions, tracking and evaluation
• Ensure effective follow-up process and prompt communication to applicants
• Confirm that a link to the application process is available on all country NMRA websites
• Create a clear ECOWAS-MRH process pathway for applicants to follow
• Include a drastic reduction in country good manufacturing process (GMP) fees for sites already evaluated through the process
• Discourage the need for a new set of GMP fees for each NMRA, as this will have great impact on the ability to progress the application in each ECOWAS country
• Concentrate the ECOWAS-MRH initiative solely on the quality and efficacy of the products, as decisions on products’ commercial presentation should be left to individual countries for effective cost management
• Improve communication of the multiple benefits of the ECOWAS-MRH initiative as it is presently poorly communicated among participating companies
• Strengthen the regulatory function and inspection
• Respond quickly to submitted documents, especially dossiers from applicants

## 4 Discussion

This study obtained the views of the both generic and innovator pharmaceutical applicants about the performance of the programme to date, identified the challenges experienced throughout the life cycle of the ECOWAS-MRH initiative, determined the strengths and weaknesses of the initiative, identified the ways of improving the performance of the work-sharing programme and envisaged the strategy for moving forward. The applicants expressed their views on all aspects of the ECOWAS-MRH initiative and offered very valuable suggestions for its improvement.

The important benefit of harmonisation of registration requirements across the region was identified by almost all the applicants at this time. It is noted that in a similar study of the ECOWAS-MRH initiative by the authors (Owusu-Asante et al., 2022), the benefit of harmonisation of registration requirements across the region was also highlighted by the NMRAs. Since the views of the applicants and NMRAs are the same at this time regarding this benefit as well as the benefits of the initiative to patients, it is expected that both stakeholders would work together to facilitate registration of medicines and vaccines within the ECOWAS member states, ultimately resulting in timely patient access to these products.

The medicines regulatory harmonisation initiative in West Africa (ECOWAS-MRH) aims to improve the registration timelines without compromising the quality of regulatory decisions submitted for marketing authorisation. The joint assessment procedure, which is patronised by the NMRAs in the West African region and pharmaceutical manufacturers in other parts of the world is a classic example of this harmonisation initiative (AllAfrica.com, 2012; Dansie, et al., 2019; Giaquinto et al., 2020).

As noted by the applicants, the strength of the ECOWAS-MRH initiative to prioritise the review of ECOWAS-MRH products for subsequent registration in the member states can be maximised to help make such medicines and vaccines available for public health use in a timely manner. The clearly identified benefits to applicants derived from the ECOWAS-MRH initiative (Figure 3) resulting in savings in time and resources and access to various markets at the same time provide justification to ensure that the ECOWAS-MRH initiative is supported to achieve its mandate.

Some of the challenges of the ECOWAS-MRH initiative namely, lack of centralised submission and tracking which were identified by the applicants in the study were also identified by the NMRAs in the previous study (Owusu-Asante et al., 2022). Similar to the studies conducted with regard to the EAC-MRH and ZaZiBoNa (Ngum et al., 2022; Sithole et al., 2022), these challenges were also reported with regard to the respective regional MRH initiatives. In addition, the applicants in this study identified other challenges that were specific to the ECOWAS-MRH initiative such as differences in regulatory performance of the countries and dependence on the countries’ process for communication with applicants, and lack of detailed information on the process for applicants.

The challenges faced by manufacturers submitting applications to the ECOWAS-MRH initiative such as low motivation to use the ECOWAS-MRH route and preference for other regulatory pathways in the ECOWAS member states is reflected in the low number of applicants who have accessed the ECOWAS-MRH initiative to date (Blanc, 2020). Other challenges such as lack of information on country and ECOWAS-MRH websites about the process, milestones, timelines and pending and approved products were also reported by the NMRAs (Owusu-Asante et al., 2022) and should therefore be addressed for the joint-benefit of the NMRAs and applicants.

Suggestions to improve the effectiveness of the ECOWAS-MRH initiative provided by the applicants were previously offered by the NMRAs, such as making publicly available any information that might help applicants in managing their submissions such as document templates, lists of questions and answers, timelines and milestones, decision-making transparency aids such as publishing Public Assessment Reports, consistency in application of guidelines and decisions, engagement and interaction with stakeholders, minimising the need for country-specific documents and publishing of pending and approved products. Use of risk-based approaches for example, reliance pathways was also suggested as another way that could be explored to improve the effectiveness of the ECOWAS-MRH initiative. The WHO Prequalification Programme success stories should be examined and piloted in the ECOWAS region (WHO, 2022b).

The suggestions presented by the applicants to improve the efficiency of the ECOWAS-MRH initiative, were also previously provided by the NMRAs, such as implementing robust IT systems and building capacity of assessors to facilitate processing and monitoring milestones of applications should also be explored (Owusu-Asante et al., 2022). Similar to the NMRAs, the applicants’ viewed the establishment of a regional administrative body, an ECOWAS regional medicines agency, if legally possible, to manage the ECOWAS-MRH initiative as the most progressive way forward. Therefore, the study described here details a similar evaluation by pharmaceutical industry users of this programme and identified strategies that would strengthen the ECOWAS-MRH initiative moving forward. The pharmaceutical manufacturer participants identified benefits including harmonised requirements and simultaneous accessibility for medicines and challenges including a lack of centralised submission and tracking and a preference for other regulatory pathways. Several approaches to increase its effectiveness are recommended including implementation of risk-based approaches such as use of reliance pathways; building assessor capacity to facilitate processing and monitoring applications; and priority review of ECOWAS-MRH products. Such outcomes of this study provide a unique perspective to that of the previous study assessing the views of the NMRAs in the ECOWAS region.

The views of the NMRAs from the previous study (Owusu-Asante et al., 2022) have been endorsed by the applicants and therefore should be noted. Five of the ten manufacturers who participated in the study were based in the ECOWAS region. Local manufacturers in the region should be technically and financially supported in order to encourage them to benefit from the ECOWAS initiative.

The authors’ key recommendations for improving the ECOWAS-MRH initiative for both generic and innovator applicants are.

• **Provide incentives to applicants in ECOWAS** through fast-track processing of applications and reducing GMP inspection fees to encourage more submissions from applicants to the ECOWAS-MRH initiative.
• **Engage with the WHO Prequalification Programme** to create a facilitated regulatory pathway for medicines and vaccines that have been issued with recommendation letters following successful completion of the ECOWAS-MRH joint assessment procedure.
• **Provide eligibility for international procurement agencies** to source medicines and vaccines with recommendation letters for public health use in situations where there are no prequalified alternatives
• **Encourage training for applicants** to develop their skills, knowledge, and competence
• **Convene stakeholder meetings** on a biannual basis to engage with manufacturers and update them on requirements to ensure compliance with regulations

## 5 Conclusion

This study identified the benefits and challenges of the ECOWAS-MRH initiative as experienced by the applicants as well as strategies available to improve its effectiveness and efficiency. If implemented by both generic and innovator applicants, the key recommendations that have been proposed should further strengthen this initiative to enable it to fulfil its mandate in the ECOWAS region.

## Data Availability

The original contributions presented in the study are included in the article/supplementary materials, further inquiries can be directed to the corresponding author.

## Abbreviations

CPP, certificate of pharmaceutical product; EAC-MRH, East African community- medicines regulatory harmonization; ECOWAS, economic community of West African States; GMP, good manufacturing process; IT, information technology; NMRAs, national medicines regulatory authorities; PEER, process, effectiveness and efficiency rating; WAHO, West African health organization; WHO, world health organization; ZaZiBoNa, zambia zimbabwe botswana namibia.
